# Systemic Bevacizumab for Severe Recurrent Respiratory Papillomatosis

**DOI:** 10.1155/2022/2767996

**Published:** 2022-11-29

**Authors:** Swetha Tatineni, Zachary Warren, Mark A. Applebaum, Fuad M. Baroody

**Affiliations:** ^1^Pritzker School of Medicine, The University of Chicago, Chicago, Illinois, USA; ^2^Department of Surgery, Section of Otolaryngology-Head and Neck Surgery, The University of Chicago, Chicago, Illinois, USA; ^3^Department of Pediatrics, The University of Chicago, Chicago, Illinois, USA

## Abstract

Recurrent respiratory papillomatosis (RRP) is the most common benign pediatric laryngeal neoplasm. Various adjuvant medical therapies have failed to reliably decrease surgical frequency in this challenging airway disease. Recently, systemic bevacizumab has shown promise in advanced, treatment-resistant papillomatosis. We describe the use of systemic bevacizumab in two children with severe RRP unresponsive to other therapies. Voice and breathing improved dramatically in both patients with minimal side effects. Both patients have not required surgery in 24 months and 16 months, respectively. Systemic bevacizumab is a promising long-term treatment for severe RRP, with oncology playing an important role in patient care.

## 1. Introduction

Recurrent respiratory papillomatosis (RRP), the most common benign pediatric laryngeal neoplasm, is caused by human papillomavirus (HPV) and characterized by recurrent proliferation of squamous papillomas within the airway [1]. Age at diagnosis <5 years is associated with aggressive disease, resulting in persistent voice disturbance and airway obstruction [2]. There is no cure, and surgery remains the standard of care, though up to 20% of patients require adjuvant medical therapy from having more than four surgeries per year, airway compromise, or distal multisite spread of disease [1].

Bevacizumab (Avastin, Genentech) and its biosimilar bevacizumab-awwb (Mvasi, Amgen) are recombinant humanized monoclonal antibodies that target vascular endothelial growth factor (VEGF) to inhibit angiogenesis. Despite the well-established efficacy in adult oncology, bevacizumab use remains limited in pediatrics [3, 4]. Intralesional bevacizumab injections have limited efficacy in multifocal RRP [5–8]. Recently, systemic bevacizumab has shown promise in advanced, treatment-resistant papillomatosis [8–13]. Here, we describe the long-term use of IV bevacizumab for severe RRP affecting two children.

## 2. Case 1

**Figure 1:**
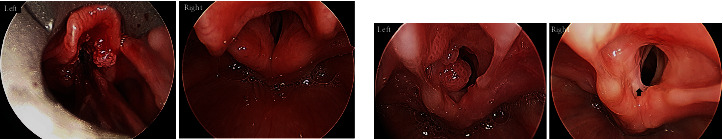
(a) Extensive papillomas along the epiglottis, bilateral aryepiglottic folds, and bilateral true and false cords with extension into the subglottis (left) and residual small papillomas on the laryngeal surface of the epiglottis after 2 months and 3 treatments of bevacizumab (right). (b) Severe laryngeal papillomas of the left larynx (left) and disappearance of disease after 3 months and 4 treatments of bevacizumab (right). There is a small scar band in the posterior glottis (black arrow).

An 8-year-old boy was diagnosed with RRP (HPV serotype 6) at 22 months and required 26 surgeries by age six. He failed adjuvant therapies, including recombinant HPV vaccine (types 6, 11, 16, and 18) and nineteen intralesional bevacizumab injections. Systemic bevacizumab-awwb treatment was planned under pediatric oncology supervision. Pretreatment direct laryngoscopy showed extensive papillomas along the epiglottis, bilateral aryepiglottic folds, and bilateral true and false cords with subglottic extension (Figure 1(a)). Trachea was spared, and chest CT showed no pulmonary disease. In June 2020, the patient underwent his first bevacizumab-awwb infusion of 10 mg/kg, ultimately receiving four treatments at three-week intervals. After the first treatment, the patient had significant improvement in voice. Direct laryngoscopy after the third treatment showed few small papillomas on the laryngeal surface of the epiglottis (Figure 1(a)). After the fourth treatment, infusions were spaced by three additional weeks after each set of three treatments. The only side effect was mild stomachache that resolved after the first treatment. As of June 2022, the patient completed his final 12-week interval treatment and remains asymptomatic with last surgery in July 2020. Infusions will be spaced to three treatments every 16 weeks before reaching a stable infusion interval every six months.

## 3. Case 2

A 7-year-old girl was diagnosed with RRP (HPV serotype 11) at 18 months and required 30 surgeries by age six. She failed adjuvant therapies, including recombinant HPV vaccine (types 6, 11, 16, and 18), six intralesional modified vaccinia Ankara E2 virus vaccine injections, and four intralesional bevacizumab injections. Systemic bevacizumab-awwb treatment was planned under pediatric oncology supervision. Pretreatment direct laryngoscopy showed extensive papillomas along the left true cord, ventricle, and false cord extending to the laryngeal surface of the epiglottis (Figure 1(b)). Trachea was spared, and chest CT showed no pulmonary disease. In February 2021, the patient underwent her first bevacizumab-awwb infusion of 10 mg/kg, ultimately receiving four treatments at three-week intervals. After the first treatment, the patient had significant improvement in voice and breathing. Direct laryngoscopy after the fourth treatment showed no papillomas and a scar band in the posterior glottis (Figure 1(b)). Subsequently, infusions were spaced as in Case 1. The only side effect was trace proteinuria that resolved after the first treatment. As of June 2022, the patient completed her final 9-week interval treatment and remains asymptomatic with last surgery in February 2021. Infusions will be spaced to 12 weeks apart with plans to continue spacing per Case 1.

## 4. Discussion

RRP is a challenging airway disease, especially in children whose disease is generally more aggressive than adults [14]. Multiple surgeries are often necessary to maintain airway patency and laryngeal anatomy, predisposing patients to increased risk of general anesthesia and surgical complications. Pediatric patients undergo an average of twenty procedures in their lifetime, posing emotional and financial costs to patients and their families [15, 16]. Many adjuvant therapies, including interferon-alfa, intralesional cidofovir, and intralesional bevacizumab, have failed to consistently decrease papilloma burden [8].

Systemic bevacizumab has been shown to reduce surgical frequency. Bevacizumab targets VEGF to inhibit angiogenesis, indirectly inhibiting papilloma growth. Papilloma epithelium and underlying vascular endothelium show strong expression of VEGF and VEGFR-1 and VEGFR-2, respectively [17]. However, underlying viral infection is unaffected, and recurrence after stopping bevacizumab is possible [9, 11, 18].

We present two children who continue to receive systemic bevacizumab-awwb, which is structurally, functionally, and clinically similar to bevacizumab [19]. Both patients received identical infusion schedules with a follow-up of 24 months and 16 months, respectively. Immediate improvement in respiratory and vocal symptoms was observed after just one treatment. Normal vocal quality often correlates with significant reduction of laryngeal lesions which was observed [13]. Both patients have remained asymptomatic with increasing treatment intervals, with the longest current interval being 16 weeks between treatments. The only side effects observed were trace self-resolving proteinuria and stomachache, consistent with prior studies demonstrating good tolerability of bevacizumab among children [4].

A recent international consensus statement for systemic bevacizumab use in RRP proposed that multiple disciplines, including pediatric oncology, are necessary, highlighting a novel treatment paradigm for this benign but aggressive airway tumor [20]. Long-term effects and appropriate dosing schedules, including treatment endpoint, are unknown. Anecdotal evidence suggests spacing treatments as detailed above. If symptoms recur, treatment frequency can be increased. Accumulated clinical experience through multidisciplinary partnerships may facilitate efforts to standardize bevacizumab administration. A national registry of patients detailing treatment schedules and response is essential to inform future therapeutic decisions and clinical trials.

## Data Availability

Data are available upon request or included within the article.

## Abbreviations

RRP: Recurrent respiratory papillomatosis
HPV: Human papillomavirus
VEGF: Vascular endothelial growth factor
VEGFR: Vascular endothelial growth factor receptor.
